# Development and Validation of the Quantification Method for Hydroxychloroquine in Volumetric Absorptive Microsampling (VAMS) Using High-Performance Liquid Chromatography-Photodiode Array

**DOI:** 10.1155/2021/3500279

**Published:** 2021-09-30

**Authors:** Yahdiana Harahap, Siti Ardyanti Rohadatul ‘Aisy, Baitha Palanggatan Maggadani

**Affiliations:** ^1^Faculty of Pharmacy, Universitas Indonesia, Depok 16424, West Java, Indonesia; ^2^Faculty of Military, Indonesia Defense University, Bogor 16810, West Java, Indonesia

## Abstract

Hydroxychloroquine is an antimalarial drug used for systemic lupus erythematosus, rheumatoid arthritis, and malaria treatment. However, hydroxychloroquine has several side effects such as ocular toxicity, neurotoxicity, gastrointestinal disorder, and also severe toxicity such as cardiotoxicity. Therefore, therapeutic drug monitoring of high dose or long-term use of hydroxychloroquine is needed. This study aims to obtain an optimum and validated analysis and preparation method for hydroxychloroquine in volumetric absorptive microsampling (VAMS) using the high-performance liquid chromatography–photodiode array detector based on the Food and Drug Administration guidelines (2018). Hydroxychloroquine quantification was performed using HPLC-PDA with Waters Sunfire™ C_18_ (5 *µ*m; 250 × 4,6 mm) column. Mobile phase consists of acetonitrile-diethylamine 1% (65 : 35, v/v) (isocratic elution) and delivered at a flow rate of 0.8 mL/min throughout the 12 minutes run. Sample in VAMS is extracted by liquid-liquid extraction with ammonia 1% and n-hexane-ethyl acetate (50 : 50 v/v) as a extraction solvent. This method has successfully qualified the Food and Drug Administration (2018) parameters, with 2 ng/mL of LLOQ, range of calibration curve 2–6500 ng/mL, and coefficient of correlation 0.9993–0.9997.

## 1. Introduction

Hydroxychloroquine is a hydroxyl analogue compound of chloroquine which is a derivate of 4-aminoquinoline. Hydroxychloroquine has antiparasitic, anti-inflammatory, and immunomodulatory, which has been used widely for the treatment and prevention of malaria, systemic lupus erythematosus (SLE), rheumatoid arthritis (RA), autoimmune conditions, and also has a role in anticancer therapy [1]. Hydroxychloroquine has also been one of the therapeutic options for COVID-19 treatment, but its use has been withdrawn because the potential risks outweigh the benefits [2, 3]. High doses or long-term use of hydroxychloroquine has potential to cause cardiotoxicity (heart rhythm disturbances), corneal toxicity, gastrointestinal disorders, hypoglycemia, and nerve cell damage [4, 5]. Therefore, a method to quantify the hydroxychloroquine level in blood is needed to determine the potential side effects that will arise.

Analysis of hydroxychloroquine and its metabolites has been done in several previous studies, both in whole blood using the venipuncture technique and VAMS itself [6–9]. Analysis using the venipuncture biosampling technique has several disadvantages such as more blood volume required, invasive, and the effects of hematocrit and homogeneity [10]. These problems have been overcome by the DBS (dried blood spot) with the finger prick biosampling technique; however, the hematocrit effect and spot area differences were still found on DBS. Thus, in recent years, various studies have been done with VAMS, designed to have the same advantages as DBS but avoids the problems of hematocrit and sample homogeneity. VAMS can collect blood with a smaller volume, accurate and precise, simpler, so it can be done at home and without medical assistance [11]. The novelties of this research are the use of the VAMS biosampling technique to simplify the sampling process and the use of HPLC-PDA which is relatively more economical than LC-MS/MS, so it is expected to be applied in the future for therapeutic drug monitoring for SLE and RA patients who receives hydroxychloroquine regimen in the hospitals.

## 2. Materials and Methods

### 2.1. Chemicals and Reagents

Hydroxychloroquine and chloroquine as the internal standard were obtained from Sigma Aldrich (United States of America). Whole blood was obtained from Indonesian Red Cross (Jakarta, Indonesia), and volumetric absorptive microsampling was obtained from Neoteryx® (Torrance, CA, United States of America). Methanol and acetonitrile are HPLC grade and were obtained from Merck Co. Ltd. (Darmstadt, German) along with diethylamine, triethylamine, ammonia, n-hexane, and ethyl acetate. Aquabidest used as a solvent was obtained from Ikapharmindo (Jakarta, Indonesia).

### 2.2. Instrumentation

High-performance liquid chromatography system consists of the LC-20AD pump (Shimadzu, Japan), autosampler SIL-20A (Shimadzu, Japan); C_18_ Column Sunfire™ (5 *µ*m; 250 × 4,6 mm) (Waters, United States of America), photodiode array detector Waters2996 (Waters, United States of America), and data processor on the computer (Dell, United States of America). The column temperature was controlled at 45°C. Mobile phase consists of acetonitrile-diethylamine 1% (65 : 35, v/v) (isocratic elution) with the injection volume of 100 *µ*L and delivered at a flow rate of 0.8 mL/min throughout the 12 minutes run.

### 2.3. Preparation of Stock Solutions, Working Solutions, Calibration Standards, and Quality Control Samples

The stock solution hydroxychloroquine and chloroquine was prepared by dissolving the standard with aquabidest as a solvent to obtain a concentration of 1000 ppm (1000 *µ*g/mL). Working solutions were prepared by serial dilutions in aquabidest to achieve a concentration of 1 ppm. All stock and working solutions were stored in the refrigerator at 4–8°C. The working solutions are also prepared for calibration standard and quality control (QC) samples by diluting a volume of stock solution in order to obtain a concentration of a hundred times the calibration standards and QC samples. To achieve concentrations of calibration standard and QC samples, the working solution spiked to drug-free whole blood with spiking ratio 1 : 100. 10 *µ*L working solution was added to 990 *µ*L drug-free whole blood to achieve 1000 *µ*L of spiked blood. The calibration standards were prepared at concentrations of 2, 10, 50, 100, 500, 1000, 3000, and 6500 ng/mL, whereas for quality control-low (QCL), quality control-medium (QCM), and quality control-high (QCH) were prepared at concentrations of 6, 2600, and 4875 ng/mL, respectively. Calibration standards and QC samples were freshly prepared for each analytical run.

### 2.4. Sample Preparation and Extraction Procedure

Calibrations standards and QC samples were prepared by dipping the tip of VAMS samplers into the spiked blood, and VAMS absorbed 30 *µ*L of spiked blood. Dipped VAMS devices were positioned in dedicated rack for being air-dry for 2 hours at room temperature. Dried VAMS were extracted by removing the tips from plastic handles and put them into microtubes. 30 *µ*L of 1 ppm internal standard (IS) working solutions and 500 *µ*L of 1% ammonia solutions were added into the microtube. The tubes were mixed on the vortex for 15 secs and continued with sonication for 5 min. Then, 500 *µ*L of n-hexane-ethyl acetate (50 : 50, v/v) was added into the tubes without taking out the tips. The tubes were mixed on the vortex for 15 s and centrifuged at a speed of 10,000 rpm for 5 min consecutively. After centrifugation, the mixture was divided into two phases. The n-hexane-ethyl acetate phase transferred into new tubes. The solvents were evaporated under the nitrogen gas flow, and the residues were reconstituted with 150 *µ*L of mobile phase. After slight sonicating and mixing with the vortex, the solution was injected into the HPLC-PDA system for analysis [12, 13].

### 2.5. Validation Procedure

#### 2.5.1. Lower Limit of Quantification (LLOQ)

LLOQ was determined by analyzing five replicas of samples and blank. The LLOQ concentration must be greater than 5 times the blank response and has %diff less than ±20% as well as %CV less than 20%. If the tested concentration has met the requirements, then analysis of half of the concentration can be carried out.

#### 2.5.2. Calibration Curve

Calibration curve was determined by analyzing minimum of 6 concentrations, blank, and zero sample. Each concentration was analyzed three times. The linear equation was made by plotting the PAR (peak area ratio) of analyte to the IS versus nominal concentrations. The calibration standard concentration recalculated by the linear equation must be within ±15% of the nominal concentration, except for LLOQ, it must be within ±20% of the nominal concentration. At least 75% of the calibration standards must meet this criterion and LLOQ must be included.

#### 2.5.3. Selectivity

The selectivity was determined by analyzing two replicas of LLOQ, blanks, and zero samples from six different blood sources. The selectivity is acceptable when blanks and zero samples are free from interference at the retention time of analyte and internal standard. The confounding component was acceptable if the response is less than 20% of the LLOQ response and less than 5% of the standard response.

#### 2.5.4. Accuracy and Precision

Accuracy and precision were determined by analyzing within-run and between-run of LLOQ, QCL, QCM, and QCH. Within-run was done by analyzing minimum of 5 replicas per concentration in single run, while between-run accuracy was done by analyzing at least 5 replicas in 3 runs at least 2 different days. For accuracy, the mean concentration must be within ±15% of nominal concentration, except for LLOQ, it must be within ±20% of nominal concentration. %CV for both within-run precision and between-run precision must be less than 15%, except for LLOQ, it must be less than 20%.

#### 2.5.5. Recovery

Recovery was determined by comparing the extracted samples with corresponding extracts of blanks spiked with the analyte postextraction for three replicas of QC samples. Recovery did not have to be 100%, but must be reproducible with CV ≤ 15%.

#### 2.5.6. Carryover

Carryover was determined by analyzing the blank after analysis of a high concentration sample or ULOQ. Carryover can be accepted if the response of the analyte on the blank is not more than 20% of the LLOQ and not more than 5% of internal standard.

#### 2.5.7. Dilution Integrity

Dilution integrity was determined by spiking whole blood to obtain the concentration above ULOQ or twice the concentration of QCH. Then, the high concentration of spiked blood was diluted with drug-free whole blood to obtain a concentration of QCH and half of QCH. Analyzes were performed at least 5 replicas for each dilution factor. %diff must be within ±15% and %CV must be less than 15%.

#### 2.5.8. Stability

The stability test was carried out by analyzing the concentrations of QCL and QCH immediately after preparation and after the storage process. Short-term stability for samples and stock solution was carried out by analyzing the samples and stock solutions after storage for 0, 6, and 24 hours, while long-term stability was carried out by analyzing after storage in the refrigerator at 4–8°C for 0, 10, 20, and 30 days. Autosampler stability was carried out by analyzing immediately after inserting into the autosampler and after storing for 24 hours. Stability was accepted if %diff is less than 15% for samples or 2% for stock solutions.

## 3. Result and Discussion

### 3.1. Method Optimization

The maximum wavelength is determined by observing the absorption spectrum at 200–400 nm using the photodiode array detector integrated with HPLC. The maximum wavelength obtained for this method is 332 nm. The mobile phase chosen was acetonitrile-1% diethylamine in water (65 : 35, v/v) because it provides the optimum peak area, resolution, and tailing factor. Acetonitrile-1% triethylamine and acetonitrile-1% diethylamine-methanol were also tried as a mobile phase but did not provide better peak area and resolution. Flow rate optimization is performed to optimize the retention time of the analyte and also affects the analyte response. The peak area and resolution decrease as the flow rate increases. This is due to a decrease in the retention time of the analyte and internal standard, so that the distance between the two peaks is also smaller and causes a decrease of resolution. Therefore, a flow rate of 0.8 mL/min was chosen as the optimum flow rate rather than 1.0 or 1.2 mL/min because it provides the best peak area, resolution, and tailing factor. Although the retention time of analytes with this flow rate is longer, this is also advantageous considering that in the initial minutes, there are a lot of peaks of impurities from blood. Thus, the retention time that is too fast is also not really good considering that the analyte peaks can be disturbed by impurity peaks. Column temperature did not have a significant effect on peak area, resolution, HETP, and tailing factor. However, the column pressure decreases as the column temperature increases; this affects the column effectiveness during analysis. The column temperature increases from 25°C to 45°C causes column pressure decreases for about 150 psi. Therefore, the column temperature of 45°C was chosen as the optimum condition.

### 3.2. System Suitability Test

**Figure 1:**
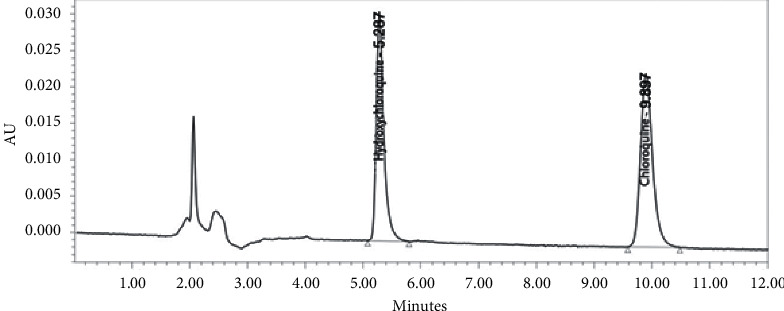
Chromatogram from the system suitability test.

From the 1 ppm of working solution analysis results, the average retention time of hydroxychloroquine was 5.29 min and the retention time of chloroquine as the internal standard was 9.91 min with total run time of 12 minutes. The mean areas obtained were 280203.5 *µ*V/s for hydroxychloroquine and 339123.5 *µ*V/s for chloroquine. From these data, it was obtained that %CV of retention time was 0.11% for hydroxychloroquine and 0.09% for chloroquine. The %CV of peak areas was 1.44% for hydroxychloroquine and 1.63% for chloroquine. The %CV for PAR itself was 0.90%. Therefore, it can be concluded that the analysis conditions used are appropriate because %CV was ≤2%. The chromatogram and data of the system suitability test are represented in Figure 1 and Table 1.

### 3.3. Optimization of Sample Preparation

Before extraction, the tips that have been spotted with blood must be dried first. The peak area of analyte increases as the drying time increases because the extraction process from dried blood is easier than undried blood. However, the response of analytes from the drying time of 2 hours did not significantly different from that of 3 hours. This means that the blood has dried completely within 2 hours. The extraction solutions were tested according to the existing extraction methods, which are protein precipitation (PP) and liquid-liquid extraction (LLE). However, the PP extraction method resulted in a smaller peak area of analyte compared to the LLE method. So, 1% ammonia solution and n-hexane-ethyl acetate (50 : 5) with a volume of 500 *µ*L each were chosen for the extraction solution for the LLE method. The advantage of the LLE method is resulting in a cleaner chromatogram compared to the PP method which has many blood impurities peaks. The peak area of analyte increases as the volume of extraction solution increases, which is related to solubility of analyte. Volume of extraction solution has also been optimized, and the volume of 500 *µ*L was able to extract analyte optimally. Mixing with vortex for 15 seconds and sonicating for 5 minutes long were chosen because at this time, it is sufficient to completely extract the analyte from the tip. Prolonging mixing and sonicating time did not give a better peak area of analyte. Centrifugation is carried out to accelerate the separation of the organic phase and the water phase. Then, the centrifugation speed and time were chosen at speed of 10.000 rpm for 5 minutes.

### 3.4. Method Validation

#### 3.4.1. LLOQ

**Figure 2:**
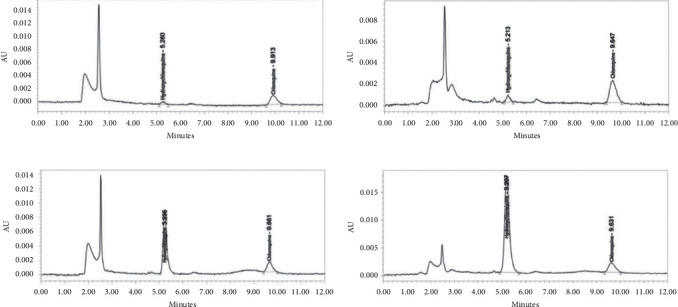
Chromatogram of (a) LLOQ, (b) QCL, (c) QCM, and (d) QCH.

The LLOQ of hydroxychloroquine obtained by this method was 2 ng/mL. %diff (the ratio of measured value and known value) was in the range −7.01–7.69%, and %CV was 10.07%. This method has a smaller LLOQ than previous studies. This is because the previous method used a protein precipitation extraction method, and the process of extracting analyte was not optimal. The chromatogram of QC samples is shown in Figure 2.

#### 3.4.2. Calibration Curve

The calibration curve used eight points of concentration, which are 2, 10, 50, 100, 500, 1000, 3000, and 6500 ng/mL, blank, and zero sample. Coefficient of correlation was in the range of 0.9993–0.9997, which means that the curve is linear and %diff of the calculated concentration was acceptable. The linear method is capable of giving well-defined results proportional to the actual analyte concentration within the specified range [14]. The data of calibration curve bot within-run and between-run are given in Table 2.

#### 3.4.3. Selectivity

**Figure 3:**
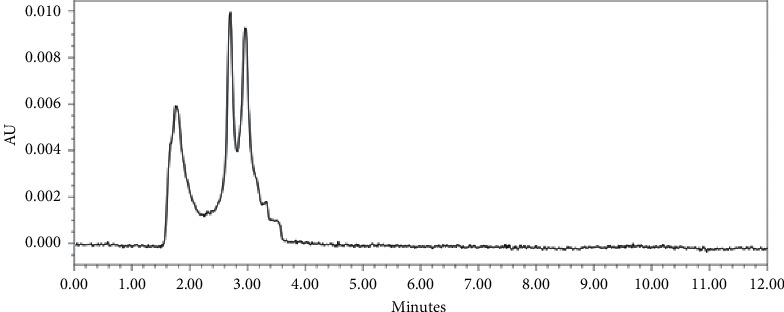
Blank chromatogram.

Blank analysis of the six blood sources did not give absorption either at the retention time of the analyte or the internal standard. Thus, the percentage of interference for each source is 0.00%. A clean blank is obtained from a good extraction process in separating the analyte and impurities from the matrix. The LLE method can provide a chromatogram that is cleaner of blood impurities compared to the protein precipitation extraction method. The chromatogram of blank and LLOQ chromatogram is shown in Figures 2 and 3.

#### 3.4.4. Accuracy and Precision

All the data have met the requirements %diff within ±20% for LLOQ and ±15% for QC samples as well as %CV less than 20% for LLOQ and 15% for QC samples. Both within-run and between-run for accuracy and precision have met those requirements. Accuracy and precision data both within-run and between-run are given in Table 3. The chromatogram of QC samples is shown in Figure 2.

#### 3.4.5. Recovery

%recovery from this method is better than the existing method, which is in the range 88.93–90.33%. %recovery can be improved by selecting the appropriate extraction process and the appropriate extraction solvent. A suitable extraction process will extract the analyte optimally that lead to good recovery.

#### 3.4.6. Carryover

None of blank analysis after ULOQ injection give any absorption in both analyte and internal standard retention time. Therefore, carryover from this method is 0.00%. This means that high concentration injection would not affect the next injection. The optimum analysis method provides a very small carryover without the needs for a longer washing step. Carryover can be influenced by the selection of the mobile phase, the particle size, and column length, as well as the autosampler performance [15]. The cause of carryover is that there are residual from previous injections left in the injection path, so that they are carried away during the next injection. Injection of high concentrations of samples causes column overload. The high affinity of the analyte to the column also makes it difficult for the analyte to elute completely. Therefore, a suitable mobile phase is needed to be able to elute the analyte optimally [16]. The blank chromatogram is represented in Figure 3.

#### 3.4.7. Dilution Integrity

Analysis of twice of QCH, QCH, and half of QCH results in %diff within ±15% and %CV less than 15%. The %CV of the concentrations of 2 times QCH, QCH, and ½ QCH was 9.23%, 9.21%, and 4.46%, respectively. Thus, it can be concluded that this method has fulfilled the dilution integrity requirements which means that dilution would not affect accuracy and precision.

#### 3.4.8. Stability

From the stability test, the stock solution of hydroxychloroquine and chloroquine is stable with for 24 hours storage at room temperature and only stable for 10 days in the refrigerator at 4–8°C. After 10 days, it is better to make a new stock solution. For the samples, it stable for 30 days at room temperature and stated to be unstable in the refrigerator at 4–8°C. The tips that stored in the refrigerator cause the blood too frozen, so the extraction process becomes more difficult. So, it is preferable to store VAMS at room temperature rather than in the refrigerator. The extracted solution stored in the autosampler is stable for 24 hours.

Based on the results of this research, the method developed has met all the validation parameters according to the Food and Drug Administration, 2018. This method is more selective than previous studies of hydroxychloroquine, with the lowest LLOQ. Apart from the advantages of VAMS compared to venipuncture and DBS that have been described above, storage of VAMS at room temperature also demonstrated stable analyte results up to 30 days, and this is a useful advantage especially for sample shipping cost.

## 4. Conclusion

The method has met all the full validation parameters according to the Food and Drug Administration, 2018. LLOQ for hydroxychloroquine was 2.0 ng/mL, and the range of the calibration curve was 2–6500 ng/mL with a correlation coefficient of 0.9993–0.9997. This method can be applied for therapeutic drug monitoring in SLE and RA patients who are taking hydroxychloroquine as a therapeutic regimen and can be used for the pharmacokinetics study of hydroxychloroquine in healthy subjects.

## Figures and Tables

**Table 1:** Data of the system suitability test.

Replicate	Retention time		Area (*µ*V/s)		
	HCQ	CQ	HCQ	CQ	PAR
1	5.28	9.90	280356	343842	0.81536
2	5.29	9.90	285879	346377	0.82534
3	5.28	9.91	282229	340734	0.82830
4	5.29	9.90	280504	337627	0.83081
5	5.29	9.91	278538	332918	0.83666
6	5.30	9.92	273715	333243	0.82137
Mean	5.29	9.91	280203.50	339123.50	0.82631
SD	0.01	0.01	4032.42	5528.10	0.00744
CV (%)	0.11	0.09	1.44	1.63	0.90

**Table 2:** Data of calibration curve of HCQ.

Concentration (ng/ml)	0.00	2.00	10.00	50.00	100.00	500.00	1000.00	3000.00	6500.00	Slope	Intercept	*r*
Replicate	Measured concentration (ng/ml)											
1	0	2.14	9.45	51.76	111.46	561.40	913.73	3095.51	6464.28	0.00142	0.06763	0.9997
2	0	1.91	9.67	49.22	105.13	555.91	917.22	3154.84	6436.90	0.00138	0.09504	0.9995
3	0	1.99	10.27	48.62	96.28	535.52	947.38	3194.42	6415.70	0.14250	0.00162	0.9993

Mean (ng/mL)	0.00	2.01	9.80	49.87	104.29	550.94	926.11	3148.26	6438.96			
SD	0.00	0.12	0.42	1.67	7.62	13.64	18.50	49.78	24.35			
CV (%)	0.00	5.90	4.31	3.34	7.31	2.48	2.00	1.58	0.38			

**Table 3:** Data of accuracy and precision, within-run, and between-run.

Nominal concentration (ng/mL)	Day	Within-run			Between-run	
		Mean measured concentration (ng/mL)	CV (%)	% difference (range)	Mean measured concentration (ng/mL)	CV (%)
LLOQ (2.00)	1	1.97	10.07	−17.01–7.69	1.97	9.24
	2	1.98	11.42	−14.20–16.67		
	3	1.97	8.17	−11.91–8.79		

QCL (6.00)	1	5.71	6.15	−9.67–5.92	5.92	7.15
	2	5.91	8.30	−11.19–7.42		
	3	6.15	6.12	−3.66–10.11		

QCM (2,600)	1	2632.53	7.19	−5.15–11.50	2605.69	8.12
	2	2434.37	6.92	−12.02–3.57		
	3	2750.18	6.17	−0.98–14.06		

QCH (4,785)	1	5110.59	5.97	−4.45–12.95	4903.42	9.46
	2	4504.87	7.82	−12.86–4.78		
	3	5094.81	9.58	−12.19–13.18		

## Data Availability

The clinical data used to support the findings of this study are included within the article.
